# Illuminating radiogenomic signatures in pediatric-type diffuse gliomas: insights into molecular, clinical, and imaging correlations. Part I: high-grade group

**DOI:** 10.1007/s11547-025-02078-9

**Published:** 2025-08-25

**Authors:** Ryo Kurokawa, Akifumi Hagiwara, Daiju Ueda, Rintaro Ito, Tsukasa Saida, Maya Honda, Kentaro Nishioka, Akihiko Sakata, Masahiro Yanagawa, Koji Takumi, Seitaro Oda, Satoru Ide, Keitaro Sofue, Shunsuke Sugawara, Tadashi Watabe, Kenji Hirata, Mariko Kawamura, Mami Iima, Shinji Naganawa

**Affiliations:** 1 Department of Radiology, Graduate School of Medicine, The University of Tokyo, 7-3-1 Hongo, Bunkyo-Ku, Tokyo, 113-8655 Japan; 2 Department of Radiology, Juntendo University School of Medicine, Tokyo, Japan; 3 Department of Artificial Intelligence, Graduate School of Medicine, Osaka Metropolitan University, Osaka, Japan; 4 Department of Radiology, Nagoya University Graduate School of Medicine, Nagoya, Japan; 5 Department of Radiology, Institute of Medicine, University of Tsukuba, Tsukuba, Japan; 6 Department of Diagnostic Radiology, Kansai Electric Power Hospital, Osaka, Japan; 7 Department of Radiation Oncology, Graduate School of Medicine, Hokkaido University, Sapporo, Japan; 8 Department of Diagnostic Imaging and Nuclear Medicine, Kyoto University Graduate School of Medicine, Kyoto, Japan; 9 Department of Radiology, Osaka University Graduate School of Medicine, Osaka, Japan; 10 Department of Radiology, Graduate School of Medical and Dental Sciences, Kagoshima University, Kagoshima, Japan; 11 Department of Diagnostic Radiology, Graduate School of Medical Sciences, Kumamoto University, Kumamoto, Japan; 12 Department of Radiology, University of Occupational and Environmental Health, School of Medicine, Kitakyushu, Japan; 13 Department of Radiology, Kobe University Graduate School of Medicine, Hyogo, Japan; 14 Department of Diagnostic Radiology, National Cancer Center Hospital, Tokyo, Japan; 15 Department of Diagnostic Imaging, Graduate School of Medicine, Hokkaido University, Sapporo, Japan

**Keywords:** Pediatric-type, Diffuse glioma, Diffuse midline glioma, Diffuse hemispheric glioma, Infant-type hemispheric glioma, Radiology-molecular correlation

## Abstract

Recent advances in molecular genetics have revolutionized the classification of pediatric-type high-grade gliomas in the 2021 World Health Organization central nervous system tumor classification. This narrative review synthesizes current evidence on the following four tumor types: diffuse midline glioma, H3 K27-altered; diffuse hemispheric glioma, H3 G34-mutant; diffuse pediatric-type high-grade glioma, H3-wildtype and IDH-wildtype; and infant-type hemispheric glioma. We conducted a comprehensive literature search for articles published through January 2025. For each tumor type, we analyze characteristic clinical presentations, molecular alterations, conventional and advanced magnetic resonance imaging features, radiological-molecular correlations, and current therapeutic approaches. Emerging radiogenomic approaches utilizing artificial intelligence, including radiomics and deep learning, show promise in identifying imaging biomarkers that correlate with molecular features. This review highlights the importance of integrating radiological and molecular data for accurate diagnosis and treatment planning, while acknowledging limitations in current methodologies and the need for prospective validation in larger cohorts. Understanding these correlations is crucial for advancing personalized treatment strategies for these challenging tumors.

## Introduction

Pediatric central nervous system (CNS) tumors represent a substantial health concern, being the most common solid tumors and the leading cause of cancer-related mortality in children and adolescents, with an incidence of 6.23 per 100,000 population [1]. Historically, like adult cases, the diagnosis of pediatric CNS tumors primarily relied on morphological characteristics. However, recent rapid advances in molecular genetics have led to the discovery of numerous important genetic and chromosomal alterations. Furthermore, molecular genetic features have demonstrated superior correlations with patient age, tumor location, treatment response, and prognosis compared to traditional morphology-based diagnostics. The WHO Classification of Tumors of the Central Nervous System underwent a paradigm shift from its morphology-focused 4th edition in 2007 to the revised 4th edition in 2016, which prioritized molecular genetic characteristics [2]. The latest 5th edition (WHO CNS5) published in 2021 further refines and advances the molecular genetic-based classification of CNS tumors through various changes, including, among others, the introduction of DNA methylation-based diagnosis, categorization of diffuse gliomas into pediatric and adult types, and incorporation of 22 new tumor types [3–6].

Clinicians treating pediatric brain tumors have long recognized the existence of tumor groups that, despite their morphological similarities to adult cases, demonstrate markedly different clinical presentations from their adult counterparts. The identification of distinctive molecular genetic features has illuminated these underlying differences [6, 7]. The classification of diffuse gliomas into pediatric-type and adult-type in WHO CNS5 represents a landmark change reflecting this knowledge [6]. Pediatric-type diffuse gliomas are now categorized into four high-grade types (diffuse midline glioma, H3 K27-altered [DMG-H3K27a]; diffuse hemispheric glioma, H3 G34-mutant [DHG-H3G34m]; diffuse pediatric-type high-grade glioma, H3-wildtype and IDH-wildtype [pHGG]; and infant-type hemispheric glioma [IHG]; Table 1) and four low-grade types (diffuse astrocytoma, MYB- or MYBL1-altered; angiocentric glioma; polymorphous low-grade neuroepithelial tumor of the young; and diffuse low-grade glioma, MAPK pathway-altered). These eight types of pediatric-type diffuse gliomas include six newly incorporated types and one renamed type, organizing multiple key diagnostic genes, molecules, and pathways.

**Table 1:** Radiological characteristics of pediatric-type high-grade gliomas

Tumor type	CNS WHO grade	Common age/location	General radiological findings	Advanced imaging findings
Diffuse midline glioma, H3 K27-altered	4	- Primarily in children, adolescents, and young adults (peak at ages 6–9) - Located in midline structures (pons, thalamus, spinal cord)	- Often large, circumscribed - Peritumoral edema is less common - T2-FLAIR mismatch sign is frequently observed in pontine lesions - Pontine lesions: minimal enhancement, “Basilar artery wrapped sign” - Thalamic lesions: may show necrosis and moderate enhancement - Rare calcification	- DSC-MRI: frequently low rCBV (especially in pontine lesions) - DWI: often high ADC in pontine lesions - APTw imaging: high sensitivity for detecting H3K27M mutation - MRS: lower myo-inositol/Cr ratios compared to H3K27-wild-type tumors - ^18^F-DOPA PET: higher tumor/striatum ratio compared to H3K27-wild-type tumors
Diffuse hemispheric glioma, H3 G34-mutant	4	- Mostly in ages 10–29 - Typically in the cerebral hemispheres (frontal, parietal)	- Hyperdense on non-enhanced CT - Heterogeneous enhancement - Frequent hemorrhage (≥ 40%) and cystic/necrotic changes - Calcification is less common (~ 22%) - Leptomeningeal and/or ependymal contact is frequent	- DWI: often shows diffusion restriction
Diffuse pediatric-type high-grade glioma, H3-wild type/IDH-wild type	4	- Occurs in children, adolescents, and young adults - Usually located in cerebral hemispheres, followed by brainstem	- Large, often necrotic mass with heterogeneous enhancement - Hyperdense on non-enhanced CT - May present with a gliomatosis cerebri–like growth pattern (~ 82% in RTK2A subclass)	- DWI: often shows diffusion restriction - Perfusion (from adult-type reference): PDGFRA- or EGFR-amplified tumors can have increased rCBV, though data in pediatric tumors need validation
Infant-type hemispheric glioma	Not assigned	- Predominantly in infants (< 1 year old) - Affects the cerebral hemispheres	- Large, circumscribed mass - Hyperdense on non-enhanced CT - Frequent hemorrhage, necrosis - Can have cystic components with enhancing walls - Often presents with significant edema; can be rapidly growing	- DWI: shows diffusion restriction due to high tumor cellularity

CNS = central nervous system; WHO = World Health Organization; FLAIR = fluid-attenuated inversion recovery; DSC = dynamic susceptibility contrast; MRI = magnetic resonance imaging; DWI = diffusion-weighted imaging; ADC = apparent diffusion coefficient; MRS = magnetic resonance spectroscopy; DOPA = dihydroxyphenylalanine; PET = positron emission tomography; rCBV = relative cerebral blood volume

**Fig. 1:**
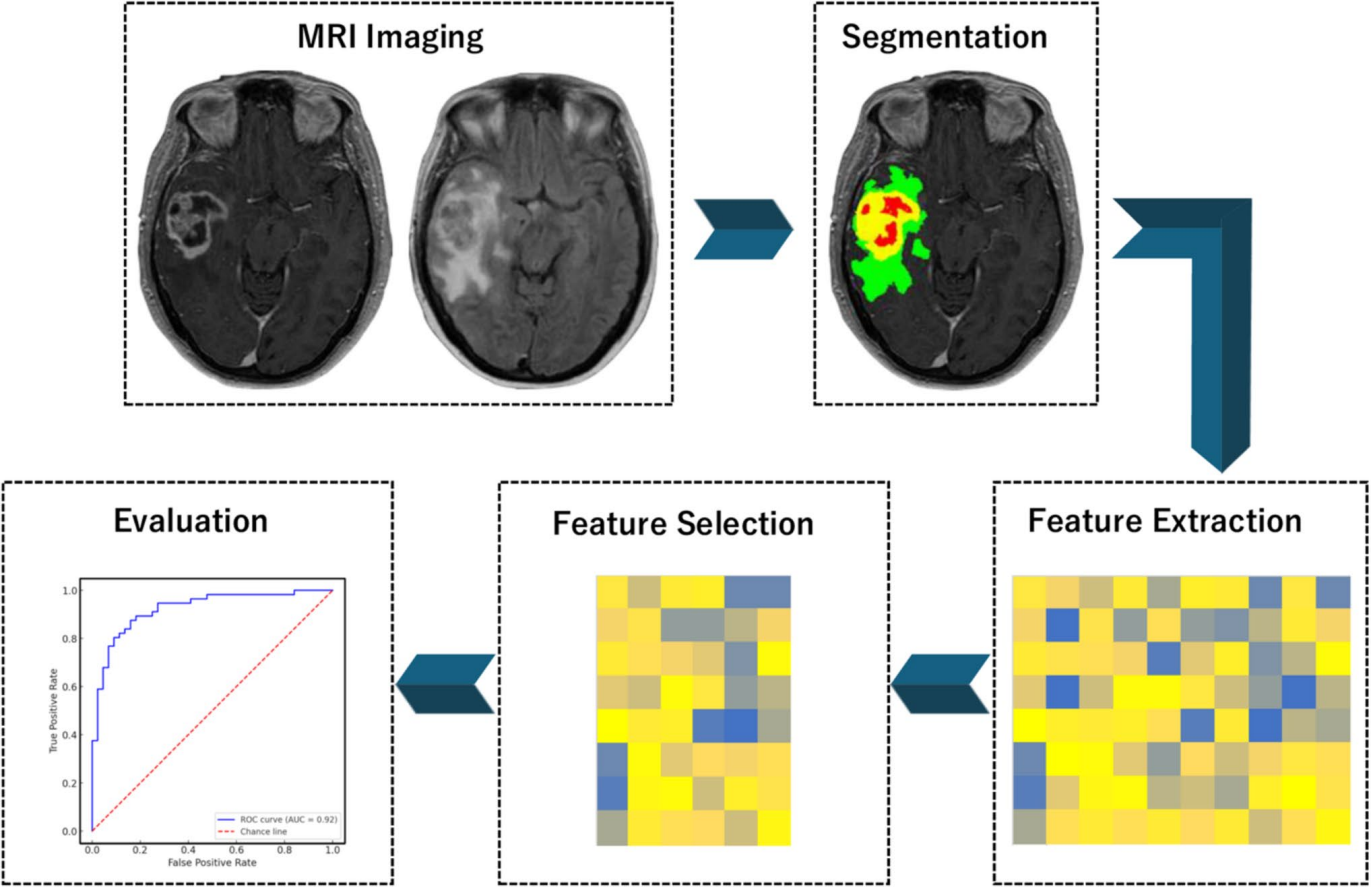
Schematic diagram of the radiomics workflow in brain magnetic resonance imaging (MRI). First, T1-weighted post-contrast and fluid attenuated inversion recovery (FLAIR) images are obtained (top left). Next, the tumor is segmented into enhancing (yellow), non-enhancing (red), and peritumoral FLAIR hyperintensity (green) regions (top right). From these segmented volumes, various quantitative radiomic features are extracted (bottom right) and then passed through feature selection (bottom center) to identify the most predictive subset. Finally, the selected features are used for model building and performance evaluation, as demonstrated by an example receiver operating characteristic curve (bottom left)

In this evolving landscape, the role of diagnostic imaging in pediatric diffuse gliomas is undergoing transformation. There is a growing emphasis on radiogenomics—the extraction of molecular genetic characteristics from imaging findings. This approach leverages not only conventional MRI sequences but also advanced sequences such as diffusion-weighted imaging (DWI), magnetic resonance spectroscopy, perfusion-weighted imaging (PWI), and chemical exchange saturation transfer techniques including amide proton transwer-weighted (APTw) imaging. Furthermore, artificial intelligence (AI) technologies are being employed to analyze vast datasets for subtle imaging patterns that may elude human detection [8, 9]. Techniques such as radiomics and deep learning not only enable the extraction of quantitative imaging biomarkers but also reveal subtle correlations with molecular genetic profiles. Radiomics is an advanced computational methodology that transforms medical images into mineable quantitative features, enabling objective and comprehensive characterization of tissue patterns beyond what is visible to the human eye (Fig. 1) [10–12]. Machine learning refers to computational algorithms that can automatically detect patterns in complex datasets and make predictions, learning from examples rather than following explicitly programmed rules [13–16]. These AI-driven integrations have become essential for precision diagnostics and personalized treatment planning in pediatric high-grade gliomas, especially in DMG-H3K27a and DHG-H3G34m.

This article presents a narrative literature review of pediatric-type high-grade gliomas. Studies were selected based on relevance, methodological quality, and publication in peer-reviewed journals, with preference given to studies published after the 2016 WHO classification revision. We review molecular, clinical, and radiological characteristics of pediatric-type high-grade diffuse gliomas, examining the relationship between imaging features on conventional and advanced MRI sequences and underlying molecular alterations. We discuss current therapeutic approaches and ongoing clinical trials, emphasizing the role of radiological-molecular correlations in improving diagnostic accuracy and treatment planning.

## Pediatric-type high-grade gliomas

Pediatric-type high-grade gliomas are a group of aggressive primary CNS tumors that primarily affect children and young adults. These tumors are characterized by their rapid growth and tendency to infiltrate surrounding brain tissue, making them challenging to treat. Historically, due to their histological similarities, pediatric-type high-grade gliomas were diagnosed and treated similarly to adult-type high-grade gliomas. However, recent evidence has revealed distinct differences in their clinical behavior, treatment response, and prognosis. Standard treatment for adult-type high-grade gliomas typically involves surgery, chemotherapy, and radiation therapy. However, these treatments are often less effective in pediatric-type high-grade gliomas [17–19].

In recent years, mutations in histone H3-coding genes (particularly K27 alterations in H3.3 and H3.1, and G34R/V mutations in H3.3) have been recognized as drivers of poor-prognosis CNS tumors by disrupting of epigenetic transcriptional regulation and causing aberrant gene expression programming. This understanding has revolutionized the diagnosis and classification of diffuse gliomas [6, 20, 21]. Furthermore, DNA methylation analysis has proven valuable for subtype diagnosis, prognostic prediction, and the identification of therapeutic targets in pediatric-type high-grade gliomas [22, 23]. Targeted therapies and immunotherapies are being explored as potential treatment options for pediatric-type high-grade gliomas.

## Diffuse midline glioma, H3 K27-altered (DMG-H3K27a)

### General

DMG-H3K27a was newly recognized in the 2016 WHO classification of the CNS tumors as "diffuse midline glioma, H3 K27M-mutant.” In WHO CNS5, this tumor is defined as a WHO grade 4 neoplasm characterized by infiltrative and midline-located glioma with loss of H3 p.K28me3, harboring either H3 K27M mutation or H3 K27M-wild type with either of EZHIP overexpression, EGFR amplification, EGFR missense mutation, or EGFR small in-frame insertion [6, 24]. In many cases, K27M mutation in one of the histone H3 genes (H3F3A (coding H3.3; approximately 70% of cases) and HIST1H3B/C (coding H3.1)) results in the substitution of lysine with methionine at position 27 of the histone H3 tail, leading to global epigenetic dysregulation and driving tumorigenesis [25, 26]. DMG-H3K27a comprises 10–15% of all pediatric brain tumors and 75% of all pediatric brainstem tumors [27].

The nomenclature change from "H3 K27M-mutant" to "H3 K27-altered" has enabled the coherent integration of tumors lacking H3 K27M mutation into the same type. However, the "midline-located" criterion excludes non-midline-located H3 K27-altered diffuse gliomas, which represent less than 5% of H3K27-altered diffuse gliomas, from the DMG-H3K27a definition [28, 29]. These non-midline-located tumors may have a more favorable prognosis compared to their midline counterparts due to their anatomically accessible location facilitating surgical resection [30]. Whether these non-midline tumors harbor identical molecular alterations to their midline counterparts remains to be elucidated.

In addition to the defining H3K27 alteration, DMG-H3K27a often exhibits other genetic alterations, including amplifications (PDGFRA (30%), CDK4/6 or CCND1-3 (20%), MYC/PVT1 (15%)), mutations (TP53 (30%), ACVR1 (30%), PPM1D (15%), ATRX (15%)), and deletions (CDKN2A/B (< 5%)) [31]. Although this tumor primarily affects children aged 6–9 and young adults aged 18–40, an improved understanding of its characteristics has led to the increased recognition of adult cases [32, 33]. The reported median overall survival ranges from 10.1 to 14.4 months after diagnosis [34].

The standard treatment consists of radiation therapy (total 54–60Gy). However, various therapeutic approaches are under development, emphasizing the importance of specific radiological diagnosis, as surgical management and biopsy are not always feasible due to the deep location of the tumors [34].

Immunotherapy approaches are being extensively studied, including H3 K27M-targeted vaccines combined with immune checkpoint inhibitors to stimulate immune responses against tumor cells. Several early-phase trials are evaluating synthetic H3 K27M peptide vaccines along with agents like nivolumab and atezolizumab. Adoptive cell therapy trials are investigating chimeric antigen receptor-T cells targeting various antigens including GD2, which is overexpressed in H3 K27M-mutant gliomas. While early results have shown some promise with radiographic improvements and clinical responses, safety concerns exist regarding inflammation in the brainstem region.

Small molecule inhibitors targeting different pathways are being evaluated, including selinexor (XPO1 inhibitor), CDK4/6 inhibitors like abemaciclib and ribociclib, and epigenetic modifiers such as histone deacetylase inhibitors [34, 35]. ONC201, a DRD2 antagonist and ClpP agonist, has shown encouraging results in recent trials with a 30% objective response rate in recurrent DMG-H3K27a [36].

Novel delivery approaches are being investigated to overcome the blood–brain barrier, including focused ultrasound to enhance the delivery of therapeutics [37]. Several trials are evaluating focused ultrasound in combination with various agents like panobinostat, etoposide and doxorubicin.

### Imaging features

**Fig. 2:**
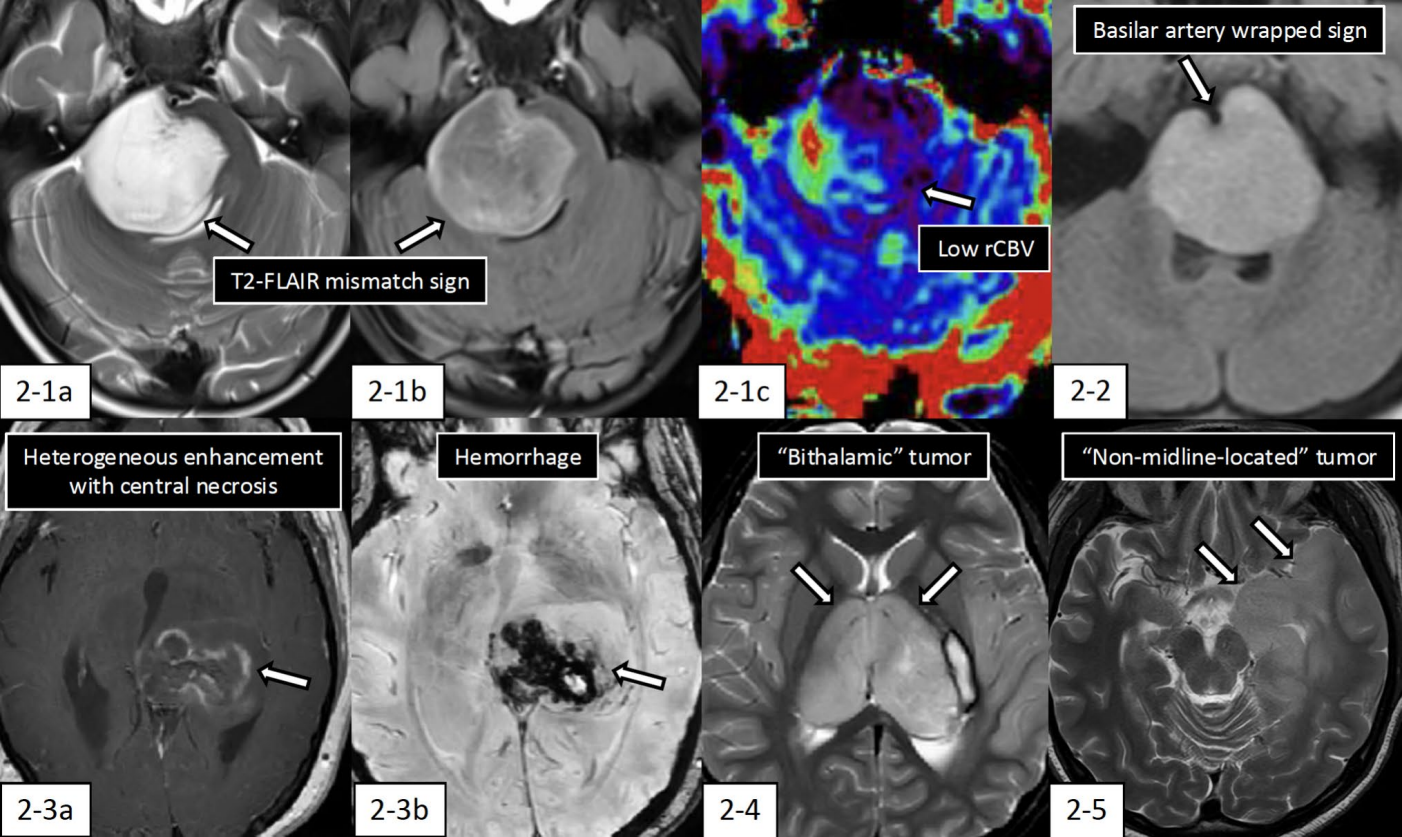
Imaging features of diffuse midline glioma, H3 K27-altered. **2-1** (4 years old, girl): Pontine tumor mass shows “T2-fluid-attenuated inversion recovery (FLAIR) mismatch sign” (2-1a and 2-1b, arrows). The relative cerebral blood volume (rCBV) shows low values in most regions of the tumor (2-1c, arrow). **2-2** (6 years old, girl): FLAIR image shows “basilar artery wrapped sign (arrow). **2-3** (36 years old, woman): The left thalamic tumor mass shows heterogeneous enhancement with central necrosis on contrast-enhanced T1-weighted imaging (2-3a, arrow) with hemorrhage shown on susceptibility-weighted imaging (2-3b, arrow). **2-4** (11 years old, girl): The “bithalamic” tumor mass is observed on fat-suppressed T2-weighted imaging (arrows). This tumor harbored H3.1K27M-mutant and an EGFR A289V mutation with an allele fraction of 56%. **2-5** (25 years old, woman): “Non-midline-located” diffuse glioma with H3K27M-mutant. The tumor mass mainly involves the left temporal lobe (arrows)

**Fig. 3:**
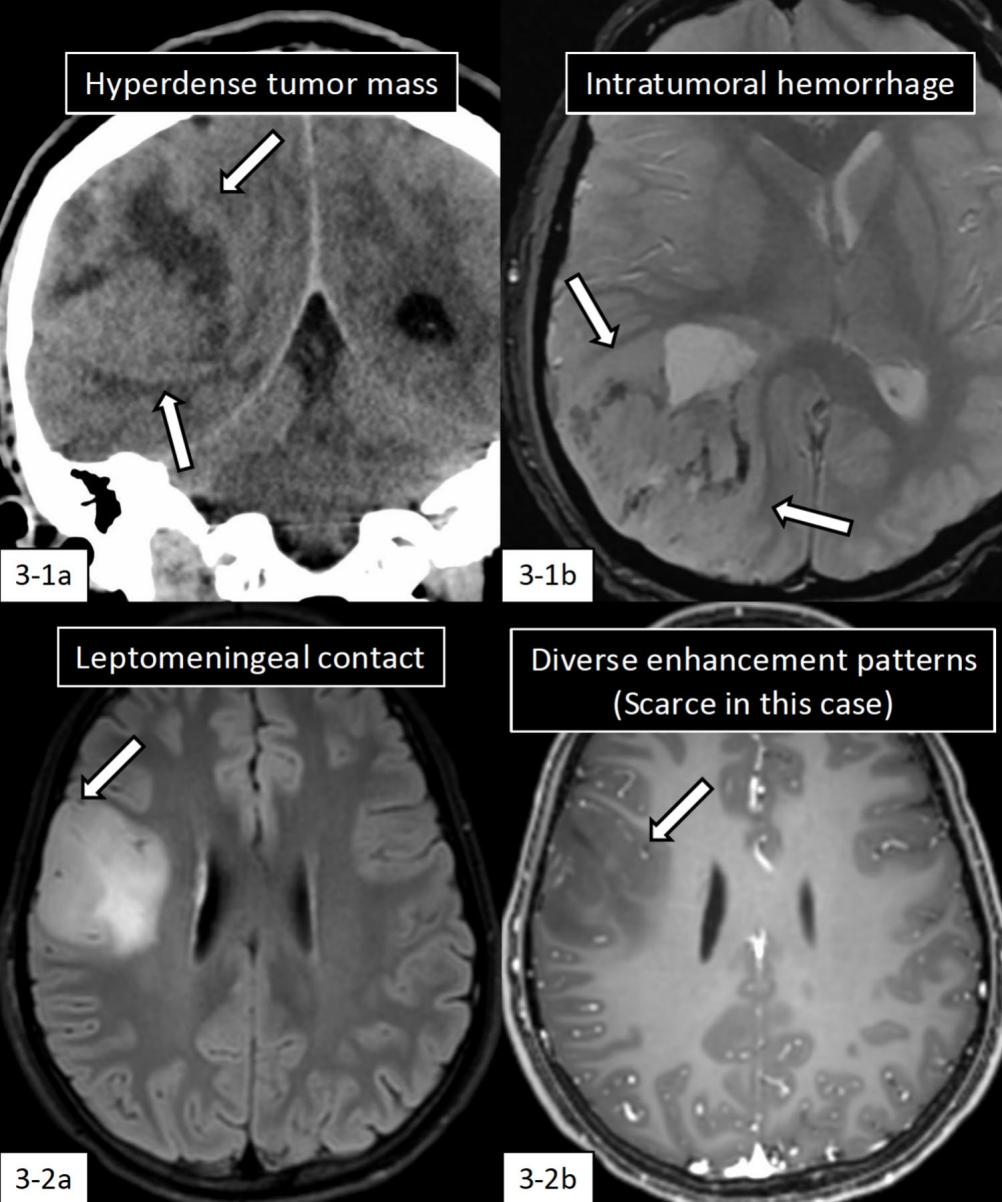
Imaging features of diffuse hemispheric glioma, H3 G34-mutant. **3-1** (22 years old, man): A large lobulated tumor mass involving the cortex with leptomeningeal contact shows hyperdensity with central necrosis on non-enhanced computed tomography coronal image (**3-1a**, arrows). Intratumoral hemorrhage is observed on T2*-weighted imaging (**3-1b**, arrows). **3-2** (16 years old, girl): A circumscribed tumor mass involving the cortex with leptomeningeal contact (**3-2a**, arrow) in the right frontal lobe shows hyperintensity on fluid attenuated inversion recovery image with a scarce contrast enhancement (**3-2b**). The same cases with different images have been evaluated in our previous study [53]

**Fig. 4:**
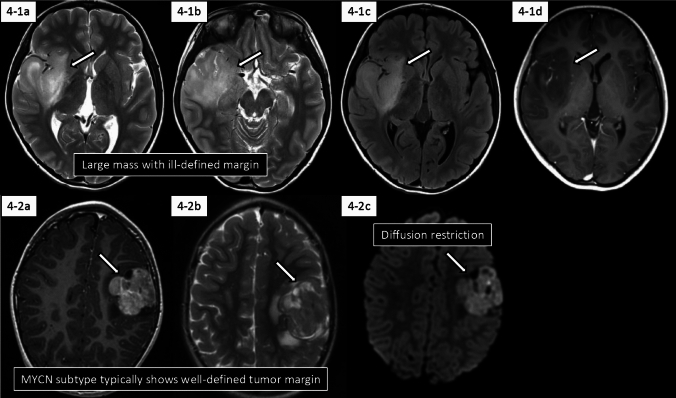
Imaging features of diffuse pediatric-type high-grade glioma, H3-wild type and IDH-wild type.** 4-1** (8 years old, boy; RTK2A subclass): There is a large tumor mass with an ill-defined margin involving the right insula and temporal lobe (arrows). The tumor mass shows hyperintensity on T2-weighted imaging (**4-1a** and **4-1b**) and fluid-attenuated inversion recovery imaging (**4-1c**) with a partial contrast enhancement (**4-1d**). **4-2** (3 years old, boy; MYCN subtype): A solid lobulated tumor mass with a well-defined margin is observed in the left frontal lobe (arrows). The tumor mass shows heterogeneous contrast enhancement on contrast-enhanced T1-weighted imaging (**4-2a**), hyperintensity with partial cystic components on T2-weighted imaging (**4-2b**), and hyperintensity on diffusion-weighted imaging (**4-2c**). The images of this case were cited from Tauziède-Espariat et al. [77] under a Creative Commons Attribution 4.0 International License

DMG-H3K27a typically presents as a large and expansive mass in midline structures (primarily thalamus, pons, and spinal cord). Approximately half of the cases demonstrate well-defined hyperintensity on T2-weighted imaging (T2WI). Calcification and peripheral edema are rare, and pontine lesions particularly tend to exhibit T2-fluid attenuated inversion recovery (FLAIR) mismatch sign (Fig. 2-1), absence or scarce contrast enhancement, low relative cerebral blood volume (rCBV), and high apparent diffusion coefficient (ADC) [38]. Pontine lesions may display a characteristic "Basilar artery wrapped sign”, where the tumor appears to engulf the basilar artery (Fig. 2-2) [39]. Thalamic lesions are more likely than pontine lesions to demonstrate necrosis, moderate to marked contrast enhancement, and low ADC values (Fig. 2-3) [40]. The formation of "bithalamic" lesions, involving both thalami, is occasionally observed and highly indicative of DMG-H3K27a (Fig. 2-4) [24]. Advanced cases may present with dissemination along the ventricular system and spinal cord surface.

Magnetic resonance spectroscopy studies have demonstrated that myo-inositol/creatine plus phosphocreatine ratios were lower than in H3K27-wild-type DMG [41]. Spinal cord DMG-H3K27a occurs predominantly in the cervical region followed by the thoracic region, with central localization within the cord [42]. Leptomeningeal thickening and enhancement are frequently observed (81%), while cystic components and hemorrhage are less common compared to H3-wild-type glioblastoma [42].

**Fig. 5:**
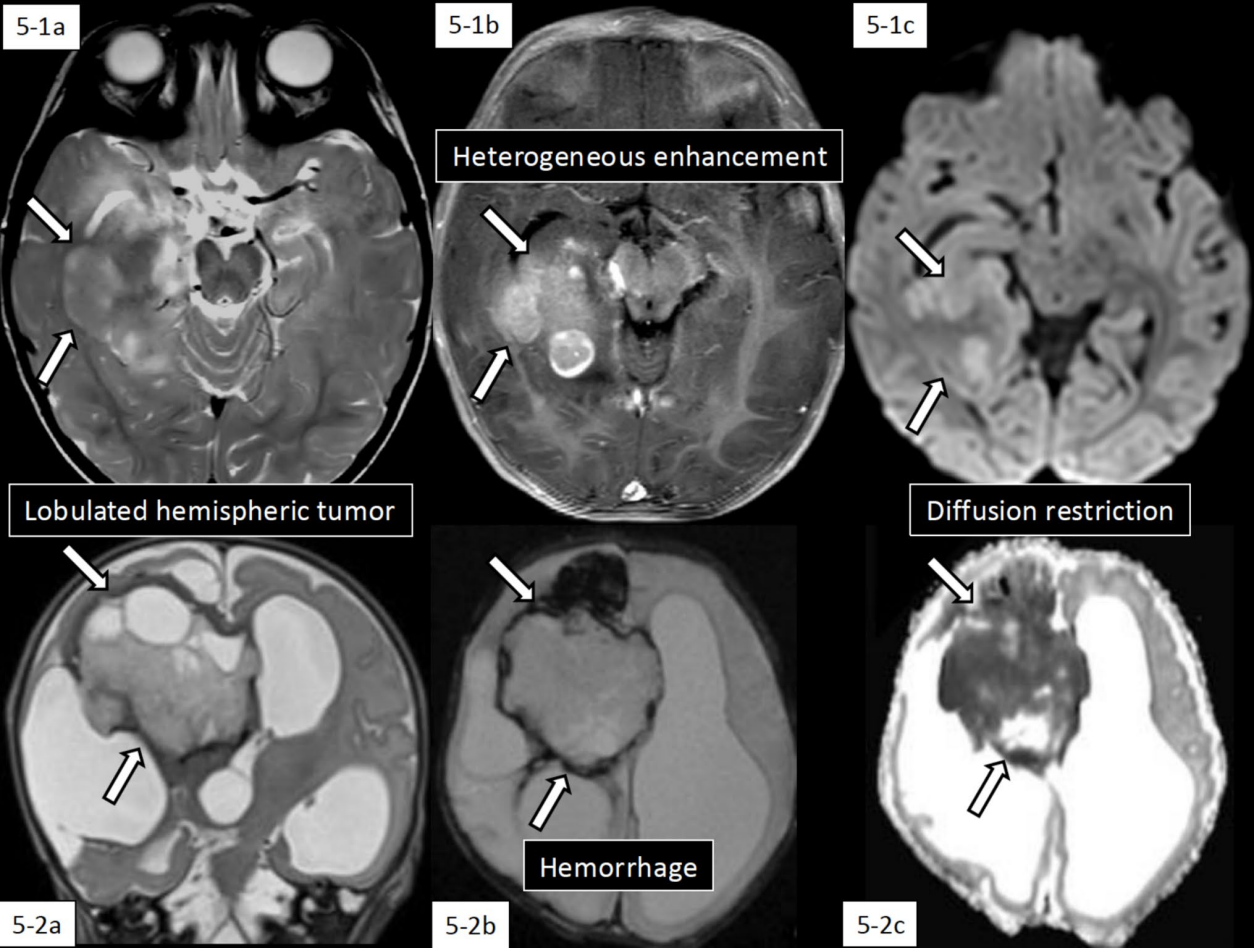
Imaging features of infant-type hemispheric glioma. 5-1 (10 months old, girl; STRN-NTRK2 fusion-positive): A lobulated tumor mass with heterogeneously hyperintense nodules on T2-weighted imaging (5-1a, arrows) and heterogeneous contrast enhancement on contrast-enhanced T1-weighted imaging (5-1b, arrows) with hyperintensity on diffusion-weighted imaging (5-1c, arrows) and low apparent diffusion coefficient (not shown). The same case with different images have been evaluated in our previous study [3]. 5-2 (a newborn; NTRK1 fusion-positive): A solid and cystic tumor mass obliterates the right foramen of Monro causing compression and contralateral displacement of the third ventricle and severe hydrocephalus (arrows). The tumor shows hyperintensity on T2-weighted coronal imaging (5-2a), hemorrhage on T2*-weighted imaging (5-2b), and low apparent diffusion coefficient indicating hypercellularity (5-2c). The images of this case was cited from Toniutti et al. [103] under a Creative Commons Attribution 4.0 International License

Although falling outside the WHO CNS5 definition as mentioned above, non-midline-located H3K27-altered diffuse gliomas most commonly arise in the temporal lobe (48%) (Fig. 2-5) [28].

### Radiology-molecular correlation

H3 K27M mutation and presumably EZHIP overexpression represent early driver mutations in DMG-H3K27a development, with H3 K27M-mutant DMG showing these alterations in nearly all cells in both primary and recurrent tumors [43]. As noted earlier, DMG-H3K27a often harbors additional genetic alterations. Consequently, DMG-H3K27a comprises a molecularly and clinically heterogeneous group of tumors with distinct DNA methylation profiles. While certain imaging characteristics common to thalamic and pontine lesions—such as low frequency of calcification, hemorrhage, and peripheral edema—appear to be features of H3 K27 alteration, DMG-H3K27a is also characterized by diverse imaging and clinical characteristics potentially associated with concurrent molecular changes.

Among DMG-H3K27a cases, those harboring concurrent MAPK pathway alterations tend to demonstrate prolonged survival (median overall survival > three years) [43–45]. Auffret et al. [43] identified a subgroup of DMG-H3K27a with co-alterations in H3 K27M and either BRAF V600E or FGFR1 mutations. The H3 K27 and BRAF/FGFR1 co-altered DMG showed significantly longer overall survival (median, 36–37 months) compared to DMG-H3K27a without co-alterations, and notably demonstrated a significantly higher frequency of macroscopic calcification. Williams et al. [46] demonstrated that H3K27/FGFR1 co-altered DMG presents in older patients (median age: 32.5 years) compared to FGFR1-wild-type counterparts, mainly arose in the thalamus (53%) and less in the pons (4.6%), with less common TP53 mutation (18.8% vs. 67%), and more common ATRX mutation (65.5% vs. 18%).

Pediatric bithalamic gliomas, characterized by symmetric thalamic enlargement in children, represent a distinct subset of DMG-H3K27a frequently exhibiting H3 K27me3 loss, H3 K27M-wild-type status, EGFR alterations (e.g., exon 20 in-frame insertions and exon 7 missense mutations), and TP53 mutations [24, 47].

Studies have been conducted to predict H3K27M mutation status from MRI and nuclear medicine imaging. Piccardo et al. [48] reported that the tumor/striatum ratio on ^18^F-dihydroxyphenylalanine positron emission tomography was statistically significantly higher in histologically high-grade H3K27M-mutant compared to H3K27M-wild-type DMG. A meta-analysis enrolling 240 cases of midline gliomas across seven studies demonstrated that H3K27M mutation status diagnosis achieved the highest sensitivity (0.92) with APTw imaging, while the combination of T1-weighted imaging (T1WI), T2WI, and contrast-enhanced T1WI showed the highest specificity (0.94) [49]. APTw imaging is hypothesized to exhibit high sensitivity due to its ability to reflect the metabolic and pathophysiological changes associated with the elevated levels of free proteins and polypeptides in gliomas with H3 K27M mutation.

AI-based methods, including radiomics and deep learning algorithms, play a pivotal role in deciphering the complex interplay between imaging features and molecular alterations. A meta-analysis of radiomics studies using machine learning (including one study utilizing deep learning [50]) encompassing 1510 midline glioma patients across 13 studies demonstrated diagnostic performance of H3K27M mutation with pooled sensitivity of 0.91, pooled specificity of 0.81, and pooled AUC of 0.84 [51].

## Diffuse hemispheric glioma, H3 G34-mutant (DHG-H3G34m)

### General

DHG-H3G34m was newly recognized in the WHO CNS5. These CNS WHO grade 4 tumors are infiltrating gliomas characterized by a specific mutation in the H3F3A gene, resulting in the substitution of glycine at position 34 with arginine or valine (G34R/V) of the histone H3.3 [6]. The H3.3 G34R/V mutation leads to decreased H3K36me3, which promotes tumorigenesis through epigenetic dysregulation-induced chromosomal instability [52]. Prior to WHO CNS5, these tumors were often diagnosed as glioblastoma or primitive neuroectodermal tumors due to their similar histological appearance. DHG-H3G34m most frequently affects patients aged 10–19 years, followed by those aged 20–29 years [53]. While the prognosis is relatively better than DMG-H3K27a, the median survival remains poor at less than two years post-diagnosis, with adult cases (> 18 years old) showing even shorter survival (median 12.8 months) [54].

Molecularly, these tumors are characterized by the loss of Olig2, an oligodendrocyte marker, with over 90% harboring TP53 and ATRX mutations, and approximately 70% exhibiting MGMT methylation [55, 56]. They are thought to originate from forebrain neural stem cells (particularly inhibitory interneuron progenitor cells) based on their retention of transcription factors that promote interneuron differentiation while suppressing oligodendrocyte differentiation (GSCX2, DLX1/2), as well as strong expression of FOXG1, which is essential for forebrain development [52].

Historically, the Stupp regimen—consisting of post-surgical temozolomide chemotherapy and radiation therapy, as established for adult IDH-wild-type glioblastoma—has been employed, though its efficacy in pediatric patients has been limited. Currently, several novel therapeutic approaches are being actively researched and developed:

In immunotherapy, phase I clinical trials are ongoing investigating dendritic cell vaccines derived from patient leukocytes in combination with nivolumab or ipilimumab [57], as well as clinical trials of neoantigen vaccines targeting tumor-specific mutations [58]. Molecular-targeted therapies under development include drugs targeting genetic mutations and amplifications in PDGFRA, BRAF, EGFR, NF1, and IDH, along with molecular-targeted therapies against CDK4/6 and Stat3 [59–61]. Substantial research efforts are focused on developing methods to effectively deliver therapeutic agents across the blood–brain barrier.

Combination therapy approaches under investigation include Ad-RTS-hIL-12 gene therapy with veledimex, AdV-tk gene therapy combined with valacyclovir and radiation therapy, and chemoimmunotherapy integrating conventional chemotherapy with immunotherapy [59]. Laemmerer et al. [62] reported that H3G34R-mutant, ATRX-loss DHGs use a mechanism called alternative lengthening of telomeres to maintain their telomeres, which is essential for cancer cell survival. They discovered that H3G34R-mutant, ATRX-loss DHGs were particularly vulnerable to combination therapy using PARP inhibitors (like niraparib) with topoisomerase inhibitors (like topotecan) [62].

### Imaging features

DHG-H3G34m occurs almost exclusively in the cerebral hemispheres, with a predilection for the frontal and parietal lobes. The tumors typically appear hyperdense on CT, while on MRI they demonstrate hyperintensity on T2WI/FLAIR, hypointensity on T1WI, and diffusion restriction (Fig. 3-1). Contrast enhancement is observed in approximately 80% of cases with diverse enhancement patterns [53, 63]. Intratumoral hemorrhage (42.2%) and cystic/necrotic changes (49.1%) are relatively frequent findings, while calcification (22.2%) is observed in a minority of cases.

While most tumors present as solitary masses, approximately 10% manifest as multifocal lesions. Leptomeningeal and/or ependymal contact is observed in the large majority of the cases (Fig. 3-2). Tumors with ill-defined borders or midline involvement have been reported to carry a poorer prognosis compared to those lacking these imaging features [53, 63].

### Radiology-molecular correlation

While H3.3 G34R/V mutation occurs early in tumor development, immunohistochemistry of H3.3G34R has revealed considerable intratumoral heterogeneity in both mouse and human gliomas [64]. For such molecular changes that exhibit intratumoral heterogeneity, radiomics analysis tends to demonstrate superior performance compared to macroscopic feature assessment in tumors developing in the CNS and other parts of the body [65, 66].

Shao et al. [67] reported that radiomics features from conventional MR images predicted H3G34 mutation status with an area under the receiver operating characteristic (AUC) of 0.925, outperforming the Visually AcceSAble Rembrandt Images (VASARI) MRI feature analysis (AUC 0.843). While the VASARI feature set offers a standardized framework for the visual evaluation and classification of glioma MRI images, its reliance on subjective interpretation limits its ability to capture subtle tumor heterogeneity. In contrast, radiomics leverages advanced quantitative analyses, providing a more nuanced and objective assessment of imaging data. This highlights the potential of radiomics to complement or even surpass traditional approaches in improving diagnostic accuracy and guiding clinical decision-making in glioma management.

## Diffuse pediatric-type high-grade glioma, H3-wild type, and IDH-wild type (pHGG, H3/IDH-wt)

### General

pHGG, H3/IDH-wt is an H3-wild-type and IDH-wild-type CNS WHO grade 4 diffuse glioma with histological malignant features, mainly affecting children, adolescents, and young adults. This tumor type was newly recognized in the WHO CNS5 classification. Accurate diagnosis requires an integrated approach combining histopathological and molecular analysis, with DNA methylation profiling playing a crucial role in its subdivision into three subtypes: RTK1, RTK2, and MYCN (Table 2) [68, 69]. A recent study has identified two distinct subclasses within the RTK2 subtype, designated as RTK2A and RTK2B, which exhibit unique clinical, radiological, and molecular characteristics [69]. While specific gene alterations, namely, alterations of PDGFRA, EGFR, and MYCN, show some correlation with pHGG, H3/IDH-wt subtypes RTK1, RTK2, and MYCN, respectively, the majority of cases lack the expected alterations in *PDGFRA*, *EGFR*, or *MYCN*, underscoring the molecular heterogeneity of these tumors.

**Table 2:** Molecular, clinical, and regional characteristics of pHGG, H3/IDH-wt

Subtype	Key molecular features	Prognosis	Typical location
pHGG RTK2	EGFR alteration (50%), chromosome 7 gain (28%)/10q loss (50%), CDKN2A/CDKN2B homozygous deletions (72%), TERT promoter mutations (64%)	Best prognosis (median OS: 44 months)	Supratentorial (96%), Infratentorial (4%)
pHGG RTK2A*	EGFR alteration (61%), BCOR mutations (78%), SETD2 mutations (39%), TERT promoter mutations (37%)	Median OS: 21.2 months	Gliomatosis cerebri pattern (82%) Bithalamic involvement (23%)
pHGG RTK2B*	EGFR alteration (80%) TP53 mutations (80%), MYCN amplification (40%) ATRX loss (80%)	Median OS: 9.7 months	Gliomatosis cerebri pattern (43%), Bithalamic involvement (86%)
pHGG RTK1	PDGFRA alteration (33%)	Intermediate prognosis (median OS: 21 months)	Supratentorial (82%), Infratentorial (18%)
pHGG MYCN	MYCN amplification (50%) with ID2 co-amplification (66%), MYC amplification (8%), TP53 mutations (67%)	Worst prognosis (median OS: 14 months)	Supratentorial (86%; primarily hemispheric), Infratentorial (14%)

pHGG, H3/IDH-wt = Diffuse pediatric-type high-grade glioma, H3-wild type and IDH-wild type; OS = overall survival

*Data for pHGG RTK2A/B is based on [69], and these subclasses have not been defined in the 5th World Health Organization classification of the central nervous system tumors

[69] Tauziède-Espariat A, Friker LL, Nussbaumer G, et al. (2024) Diffuse pediatric high-grade glioma of methylation-based RTK2A and RTK2B subclasses present distinct radiological and histomolecular features including Gliomatosis cerebri phenotype. Acta Neuropathol Commun 12:176

Histologically, pHGG, H3/IDH-wt exhibits classical glioblastoma features, characterized by hypercellularity, prominent mitotic activity, microvascular proliferation, and necrosis. pHGG, H3/IDH-wt comprises approximately 40% of pediatric-type high-grade gliomas, with pHGG MYCN and pHGG RTK2 representing the predominant and least frequent subgroups, respectively [70]. pHGG MYCN, particularly cases without ID2 co-amplification, and cases harboring concomitant RB1 and SETD2 alterations, have been documented within the tumor spectrum of Li-Fraumeni syndrome, an autosomal dominant cancer predisposition syndrome caused by germline TP53 mutations [71, 72]. Conversely, Kibe et al. [73] reported three cases of pHGG, H3/IDH-wt in adult Li-Fraumeni syndrome patients, all classified as pHGG RTK1 with PDGFRA amplification. According to WHO CNS5, pediatric gliomas arising in the context of Lynch syndrome and constitutive mismatch repair deficiency also predominantly belong to pHGG RTK1 [6]. Notably, the majority of radiation-induced pediatric gliomas demonstrate histologically high-grade features and H3/IDH-wild-type status with PDGFRA alterations and CDKN2A/B loss, exhibiting molecular profiles similar to pHGG RTK1 or RTK2 [74, 75].

The current therapeutic paradigm for pHGG, H3/IDH-wt encompasses a multimodal approach incorporating surgical resection, chemotherapy, and radiation therapy, with radiation typically deferred in patients under three years of age. While temozolomide remains the primary chemotherapeutic agent, its efficacy is notably limited due to the low frequency of MGMT promoter methylation in pHGG, H3/IDH-wt. Current research in advanced therapeutic strategies predominantly focuses on targeting the RTK-PI3K pathway and modulating MYCN signaling as promising therapeutic interventions [76].

### Imaging features

pHGG, H3/IDH-wt predominantly occurs in supratentorial regions, particularly the cerebral hemispheres, though it can also develop in midline structures. Similar to adult-type IDH-wild-type glioblastoma, pHGG, H3/IDH-wt typically forms a hyperdensity on non-enhanced CT and diffusion restriction, indicating hypercellularity with necrosis and demonstrates a propensity for large tumor formation with frequent gliomatosis cerebri growth patterns (Fig. 4) [69].

Current radiological characterization of specific subtypes is primarily derived from limited case series analyses. Given that many cases were previously diagnosed as glioblastoma/high-grade glioma prior to WHO CNS5, retrospective molecular genetic analyses of cases may provide additional insights.

### Radiology-molecular correlation

Within the proposed pHGG RTK2 subclasses, RTK2A and RTK2B demonstrate distinctive imaging patterns: Infiltrative gliomatosis cerebri growth patterns are observed in 82% and 43% of cases, respectively, while bithalamic involvement is present in 23% and 86% of cases [69].

Conversely, pHGG MYCN typically presents with well-defined borders abutting the meninges, exhibiting slight perilesional edema and homogeneous contrast enhancement, constituting a characteristic imaging pattern [77].

In adult-type high-grade glioma, tumors with PDGFRA amplification and EGFR amplification demonstrate significantly elevated perfusion parameters, such as rCBV and relative cerebral blood flow [78, 79]. EGFRvIII-mutated glioblastomas exhibit increased rCBV and decreased ADC compared to EGFRvIII-wild-type tumors [80]. PDGFRA-amplified or mutated adult-type IDH-wild-type glioblastomas show significantly higher frequencies of corpus callosum involvement and multifocal lesions [81, 82]. PDGFRA, a cell surface receptor tyrosine kinase, promotes cell proliferation, migration, and angiogenesis upon activation [83]. EGFR amplification and the subsequent late-stage event of EGFRvIII mutation enhance intratumoral vascular growth and remodeling [84]. These molecular mechanisms likely account for the elevated perfusion metrics observed in adult-type high-grade gliomas harboring these alterations. While these findings derive from adult-type high-grade glioma studies, validation of similar patterns in pHGG, H3/IDH-wt could facilitate radiological subtype prediction.

While PDGFRA and EGFR amplifications typically demonstrate homogeneous intratumoral distribution [85, 86], EGFRvIII mutations in glioblastomas exhibit marked spatial and temporal intratumoral heterogeneity [87, 88]. To effectively characterize this heterogeneity, spatial transcriptomics technology with unsupervised radiomics and deep learning, which utilize artificial neural networks to identify complex patterns in imaging data without pre-specified features, may provide optimal assessment methods [89–91]. Further investigation using these analyses in pHGG, H3/IDH-wt is warranted.

## Infant-type hemispheric glioma (IHG)

### General

IHG represents a high-grade cellular astrocytoma occurring in early childhood, primarily affecting cerebral hemispheres. Its defining characteristics include either receptor tyrosine kinase (RTK) abnormalities (such as fusions in NTRK family genes, ROS1, MET1, or ALK) or an IHG-aligned methylation profile [6]. While cases previously diagnosed with “congenital glioblastoma” may predominantly fall into this tumor type, the CNS WHO grade designation is not applied to this tumor type. Guerreiro Stucklin et al. [92] identified three distinct subgroups of infant gliomas through molecular analysis:

- Group 1 (hemispheric, RTK-driven): Characterized by ALK, NTRK1/2/3, ROS1, and MET alterations, predominantly high-grade (82.8%)
- Group 2 (hemispheric, RAS/MAPK-driven): Features non-BRAF RAS/MAPK-activating alterations, exclusively low-grade
- Group 3 (midline, RAS/MAPK-driven): Typically low-grade with BRAF alterations (97.4%)

The WHO CNS5-defined IHG largely corresponds to the high-grade gliomas in Group 1 [6]. While ALK gene fusions occur in both low-grade and high-grade gliomas, NTRK/ROS1/MET fusions show a stronger association with high-grade tumors. These tumors typically present in early infancy, with a median age at diagnosis of 2.8 months (range: 0–12 months) [92].

Histologically, IHGs are highly cellular astrocytic tumors, and high-grade glioma-like morphology with moderate to marked pleomorphism, endothelial proliferation, and necrosis can be observed [93]. IHGs also include tumors with ganglioglioma-like, desmoplastic infantile ganglioglioma-like, or ependymoma-like morphology. Regardless of their morphologic features, recent studies have shown that the prognosis of IHG is better than the other high-grade gliomas in older children [92, 94, 95]. In a recent study analyzing 56 children (ages 0.0–4.4 years), IHG demonstrated significantly better outcomes compared to other types of high-grade gliomas, with 5-year event-free survival and overall survival rates of 53.13% versus 0.0% (p = 0.0043) and 90.91% versus 16.67% (p = 0.00013), respectively [96]. Notably, survival rates between IHG and low-grade gliomas showed no significant difference (p = 0.95 for 5-year event-free survival and p = 0.43 for overall survival).

Molecularly, IHG frequently harbors RTK gene fusions that drive constitutive activation of growth and survival signaling pathways. ALK and NTRK family genes represent the most common fusion partners, while MET fusions occur in approximately 6–7% of cases [94].

NCCN Clinical Practice Guidelines in Oncology: Pediatric CNS Cancers v2.2025 and the 2024 European recommendations for pediatric high-grade gliomas recommend maximal safe resection followed by age-adapted chemotherapy as the first-line therapy for IHG, with avoidance of radiotherapy where possible due to the risks in very young children. Furthermore, these guidelines emphasizes that patients harboring actionable RTK fusions (e.g., NTRK, ALK, ROS1, MET) should be considered for early enrollment in molecularly targeted clinical trials rather than conventional cytotoxic chemotherapy [97, 98]. Recent basket trials have demonstrated promising responses to targeted therapies, including dasatinib (ABL kinase inhibitor) in GAB1-ABL2-fusion-positive IHG [99]; lorlatinib (ALK inhibitor) in SOX5-ALK-fusion-positive IHG [100]; capmatinib (MET inhibitor) in MET-fusion-positive IHG [101]; and larotrectinib (TRK inhibitor) in NTRK-fusion-positive IHG [102].

### Imaging features (Fig. 5)

IHG typically presents as a large, lobulated circumscribed mass in the cerebral hemispheres, characterized by hyperdensity on non-enhanced CT and diffusion restriction, reflecting high cellularity, along with heterogeneous enhancement, necrosis, and hemorrhage (Fig. 5-1) [3, 103]. These lesions are surrounded by edematous T2WI/FLAIR hyperintensity and may cause cerebral herniation.

Both IHG and desmoplastic infantile ganglioglioma/astrocytoma (DIG/DIA), a CNS WHO grade 1 tumor, are supratentorial tumors occurring in infancy and early childhood. While they share many similar imaging characteristics, differentiation between these tumor types is crucial given their distinct biological malignancy potential, therapeutic approaches, and prognostic outcomes. Several features distinguish IHG from DIG/DIA. IHG frequently exhibits large tumoral cysts (50%) with enhancing walls, contrasting with non-enhancing walls in DIG/DIA [104]. Hemorrhage affecting > 25% of tumor volume is common in IHG but rare in DIG/DIA (Fig. 5-2) [96]. IHG demonstrates less fibrous stroma compared to the prominent desmoplastic component typical of DIG/DIA [93].

### Radiology-molecular correlation

In pediatric NTRK-fused gliomas, while predominantly hemispheric, secondary locations include brainstem/spinal cord (20%) and cerebellum (15%) [105, 106]. ROS1-fused gliomas rarely occur in brainstem/spinal cord (5.7%) or cerebellum (1.9%), instead predominantly affecting cerebral hemispheres or basal ganglia/thalamus [106].

Notably, both NTRK- and ROS1-fused gliomas share a distinct age-dependent pattern [105]:

- In infants (< 1 year): High-grade morphology with typically no other influential genetic alterations
- In older children (≥ 1 year): Variable morphological grades with concurrent genetic aberrations

## Limitation

We acknowledge that as a narrative review, our study selection may be subject to selection bias. The radiomics studies are limited by relatively small sample sizes and heterogeneous imaging protocols, necessitating validation in larger, multi-institutional cohorts before clinical implementation. Furthermore, the rapid progress of radiomics and deep learning pipelines in pediatric-type high-grade glioma is tempered by the following systemic hurdles. First, data standardization is limited. Heterogeneous MRI acquisition parameters and bioinformatic workflows introduce covariate shift that degrades cross-site performance, a problem repeatedly emphasized in multiparametric MRI methodology reviews [9]. Second, label noise and cohort size constrain reliability. The largest meta-analysis of radiomics for H3 K27M status (1,510 patients) reported pooled AUC 0.84 but noted substantial between-study heterogeneity and high QUADAS-2 risk-of-bias driven by retrospective single-center labeling [51]. Finally, patient privacy and algorithmic fairness limit data sharing. Consensus statements on trustworthy medical-AI now recommend federated or homomorphic-encrypted learning and mandatory demographic performance stratification to prevent inequitable care [90]. Tackling these obstacles is essential for translating promising algorithms into dependable bedside decision support for this vulnerable population.

## Conclusion

In conclusion, the molecular genetic classification of pediatric-type high-grade diffuse gliomas has transformed our understanding and management of these aggressive tumors. The integration of radiological features with molecular characteristics offers promising opportunities for non-invasive tumor characterization and treatment planning, particularly in the era of expanding molecular-targeted therapeutic options. AI-based approaches, including radiomics and deep learning, have enhanced our ability to identify imaging biomarkers correlating with molecular features, as demonstrated in DMG-H3K27a and DHG-H3G34m studies. While these techniques excel at extracting quantitative features from imaging data, the challenge of integrating and interpreting diverse multimodal datasets remains. As the field evolves, emerging technologies such as large language models offer promising capabilities for synthesizing complex radiological-pathological-molecular information, potentially facilitating real-time data interpretation and clinical decision support [107–110]. With continued advances in molecular-targeted therapies, these AI approaches will likely evolve beyond diagnosis to therapy monitoring and outcome prediction. As our understanding of tumor biology deepens, the importance of multidisciplinary collaboration between radiologists, pathologists, molecular biologists, and clinicians becomes increasingly essential for advancing personalized treatment strategies for these challenging tumors.
